# Cellulose nanofiber (CNF)–sakacin‐A active material: production, characterization and application in storage trials of smoked salmon

**DOI:** 10.1002/jsfa.9715

**Published:** 2019-04-23

**Authors:** Chiara Mapelli, Alida Musatti, Alberto Barbiroli, Seema Saini, Julien Bras, Daniele Cavicchioli, Manuela Rollini

**Affiliations:** ^1^ DeFENS, Department of Food, Environmental and Nutritional Sciences. Università degli Studi di Milano Milan Italy; ^2^ LGP2, Laboratory of Pulp & Paper Science Institut Polytechnique de Grenoble Grenoble France; ^3^ ESP, Department of Environmental Science and Policy Università degli Studi di Milano Milan Italy

**Keywords:** sakacin‐A, bacteriocin, active mat, *Listeria*, food biopreservation

## Abstract

**BACKGROUND:**

Sakacin‐A due to its specific antimicrobial activity may represent a good candidate to develop active packaging solutions for food items supporting *Listeria* growth. In the present study a protein extract containing the bacteriocin sakacin‐A, produced by *Lactobacillus sakei* Lb 706 in a low‐cost culture medium containing deproteinized cheese whey, was adsorbed onto cellulose nanofibers (CNFs) to obtain an active material to be used as a mat (or a separator) in direct contact with foods.

**RESULTS:**

The applied fermentation conditions allowed 4.51 g L^−1^ of freeze‐dried protein extract to be obtained, characterized by an antimicrobial activity of near 16 700 AU g^−1^, that was used for the preparation of the active material by casting. The active material was then characterized by infrared spectra and thermogravimetric analyses. Antimicrobial trials were carried out *in vitro* using *Listeria innocua* as indicator strain; results were also confirmed *in vivo,* employing smoked salmon fillets intentionally inoculated with *Listeria innocua*: its final population was reduced to about 2.5—3 Log cycles after 28 days of storage at 6 °C in presence of sakacin‐A, compared with negative control mats produced without the bacteriocin extract.

**CONCLUSION:**

This study demonstrates the possibility of producing an antimicrobial active material containing sakacin‐A absorbed onto CNFs to decrease *Listeria* population in smoked salmon, a ready‐to eat‐food product. © 2019 The Authors. *Journal of The Science of Food and Agriculture* published by John Wiley & Sons Ltd on behalf of Society of Chemical Industry.

## INTRODUCTION

In the last decades, bacteriocins produced by lactic acid bacteria (LAB) have received great attention due to their safe origin, since the producer organisms are generally recognized as safe (GRAS), as they are degraded by proteolytic enzymes in the human intestinal tract, thus they can be considered harmless.^1^, ^2^, ^3^ Therefore, the use of bacteriocins in food preservation may offer several benefits, such as safety and their use could reduce the need of chemical preservatives and thermal treatments on food products, thus meeting the consumers request for minimally‐processed food containing natural additives.^2^

There are two main methods of using bacteriocins into food: (i) *in situ*, by adding bacteriocins‐producing bacteria, or (ii) *ex situ* with the addition of purified or semi‐purified peptides.^2^, ^4^, ^5^ However, one of the main concerns on the application of these molecules is related to their low production yields and high purification costs.

An interesting solution for bacteriocins application to food can be represented by their delivery through active packaging materials. One of the potential and innovative ways to inhibit, reduce or retard microbial growth in food may derive from food packaging; this generation of food packaging include materials with antimicrobial properties able to prevent surface growth of microorganisms in foods.^6^

Sakacin‐A is a class IIa bacteriocin produced by the LAB *Lactobacillus sakei*; it is 41 amino acids peptide with a molecular mass of 4308 Da; as all the bacteriocins belonging to class IIa, it exerts an antimicrobial effectiveness against the causative microorganism of listeriosis, a highly fatal opportunistic foodborne infection.^7^, ^8^ In this frame, sakacin‐A has potential use as a bio‐preservative in the food area.^1^, ^5^, ^9^, ^10^ In particular, sakacin‐A, due to its specific antimicrobial activity, may represent a good candidate to develop active packaging solutions for food potentially contaminated with *Listeria* such as ready‐to‐eat fish, meat products and fresh cheese.^5^, ^11^

A variety of antimicrobial packaging systems have been studied: films have been produced incorporating the antimicrobial agent into the polymer, while others have used biopolymers as effective carriers of antimicrobial agent. 12, 13, 14, 15, 16, 17 Carriage of the active molecule can be obtained also through the coating technique, applying a layer of material much thinner than the underlying substrate; in this case the coating system allows the active molecules to be released into the food, possibly in a controlled manner.^10^ Examples of active package solutions in which bacteriocins are applied by coating have been reported by Ming *et al.*,^18^ Mauriello *et al.*,^15^ Ercolini *et al.*, 19 and La Storia *et al.* 20 Another alternative to produce active packages is the incorporation of the antimicrobial compound directly into the polymeric matrix; however, this procedure cannot be considered feasible when high processing pressure and temperature or incompatibility with the packaging material can inactivate the antimicrobial agents.^10^, ^21^ In parallel to continuous film, active mats have also been proposed, made up of materials not feasible as primary packaging, but with promising properties in terms of a rational application of the active substance.^22^

Some of the polymeric films used to produce active devices are cellulose‐based, with interesting properties like renewability, biodegradability, biocompatibility and being cost‐effective materials. Barbiroli *et al.* 23 reported the possibility of incorporating lysozyme and lactoferrin into paper containing carboxymethyl cellulose, that allowed non‐covalent binding of the positively charged proteins to the paper matrix; tests on thin cuts of raw meat also confirmed their antimicrobial effect. Saini *et al.* 17 developed a novel antimicrobial film with covalently linked nisin on surface of TEMPO oxidized cellulose nanofibers for food packaging. Espitia *et al.* 24 studied the effects of pediocin incorporation into a cellulosic packaging produced with cellulose acetate resin, determining the tensile strength at break (in MPa), load at break (in newtons) and elongation at break (%), water vapor permeability and structure. Santiago‐Silva *et al.* 25 developed and evaluated the antimicrobial effect of cellulose acetate matrix films incorporated with pediocin on the preservation of samples of sliced ham.

In the past decade, nanomaterial of cellulose has been developed, named cellulose nanofibers (CNFs), with diameters between 10 and 50 nm and lengths of several millimeters. This novel material can enhance mechanical and barrier properties when applied in packaging materials.^17^, ^26^ The use of cellulose nanocomposites in food packaging can help extend shelf‐life and enhance food quality, since they serve not only as barrier to moisture, water vapor, gases, and solutes but they can also be considered carriers of active substances such as antimicrobials.^25^

In the present study, a protein extract containing the bacteriocin sakacin‐A, produced by *Lactobacillus sakei* Lb 706 in a low‐cost culture medium, was adsorbed onto CNFs to obtain an antimicrobial material. The produced mats were then characterized to analyze the efficacy of surface modification of sakacin with the CNF. The efficacy of the resulting active package was assessed performing either *in vitro* antimicrobial tests and confirmed by *in vivo* storage trials of ready‐to‐eat smoked salmon fillets intentionally inoculated with *Listeria*.

## MATERIAL AND METHODS

### Microorganisms and maintenance

The sakacin‐A‐producing strain used in this study was *Lactobacillus sakei* DSMZ 6333 (Lb706) (DSMZ: Deutsche Sammlung von Mikroorganismen und Zellkulturen GmbH, Braunschweig, Germany) while *Listeria innocua* DSMZ 20649 was used as target strain. *Lactobacillus sakei* was maintained on MRS broth (DeMan‐Rogosa‐Sharpe, Merck K GaA, Darmstadt, Germany) while *Listeria innocua* on TSB (Tryptic Soy Broth; Merck K GaA). Media were inoculated (10% *v*/*v*) with a pre‐grown culture and incubated in stationary condition at 30 °C for *Lactobacillus sakei* and 37 °C for *Listeria innocua* for 16 to 24 h. Stock cultures of both microorganisms were stored at −80 °C in their appropriate liquid medium added with 20% (*v*/*v*) glycerol (VWR International, Leuven, Belgium). Cultures were propagated twice before use.

### Sakacin‐A production and purification

Sakacin‐A was produced growing *Lactobacillus sakei* in liquid batch cultures employing a low‐cost medium formulation containing: yeast extract (Costantino, Torino, Italy) 8 g L^−1^, meat extract (Merck K GaA) 8 g L^−1^, Tween‐80 (Merck K GaA) 0.5 g L^−1^, l‐arginine (Merck K GaA) 0.5 g L^−1^; all ingredients were dissolved in deproteinized cheese whey, kindly supplied by Latteria Soresina (Soresina, Italy). After medium sterilization, 1 mL L^−1^ of minerals and vitamins mix (sterilized by filtration) was added. The mix had the following composition: magnesium sulfate (MgSO_4_; Sigma‐Aldrich, St Louis, MO, USA) 10 g (50 mL)^−1^, manganese(II) sulfate (MnSO_4_; Sigma‐Aldrich) 1.9 g (50 mL)^−1^, tiamin (Merck K GaA) 0.01 g (50 mL)^−1^, niacin (Merck K GaA) 0.01 g (50 mL)^−1^, folic acid (Carlo Erba, Cornaredo, Italy) 0.01 g (50 mL)^−1^, pyridoxal (Sigma‐Aldrich) 0.01 g (50 mL)^−1^, pantothenic acid (BDH Chemicals, London, UK) 0.01 g (50 mL)^−1^, cobalamin (Carlo Erba) 0.01 g (50 mL)^−1^.

When *Lactobacillus sakei* growth and sakacin‐A production profile was studied, culture turbidity (OD) was measured spectrophotometrically at 600 nm every 15 min in a PowerWave™ XS2 Microplate Spectrophotometer (BioTek, Winooski, VT, USA); lag phase (in minutes) and maximum growth rate (OD min^‐1^) were determined fitting data through the DMFit software (https://browser.combase.cc/DMFit).

In order to have a masterbatch of sakacin‐A to incorporate into the active material, *Lactobacillus sakei* liquid cultures were carried out in a 14 L fermenter (Omnitec Bio, Sedriano, Milan) (7 L volume) applying the following conditions: 26 °C, 9 h incubation, no aeration, agitation speed 150 rpm, pH‐stat 4.5, inoculum 5% (*v*/*v*) of a pre‐grown culture in MRS medium. Cell‐free supernatant containing sakacin‐A was obtained at the end of incubation by centrifuging the culture broth at 8000 rpm for 40 min at 4 °C (Beckman Coulter, Brea, CA, USA).

Sakacin‐A was precipitated from the obtained supernatant employing ammonium sulfate (400 g L^−1^);^28^ after 1 h at 4 °C, the sample was centrifuged at 8000 rpm for 40 min at 4 °C; precipitate was dissolved (10×) in deionized water and subsequently freeze‐dried overnight (Edwards Minifast MFD 01 lyophilizer, Burgess Hill, UK).

The protein extract containing sakacin‐A (hereafter sakacin‐A extract), was characterized in terms of antimicrobial activity, evaluated against *Listeria innocua* by agar diffusion assay as reported later, and in terms of protein content, determined by Lowry assay.^27^

### Sakacin‐A extract antimicrobial activity

Sakacin‐A extract antimicrobial activity was determined employing the agar diffusion assay: aliquots of culture broth were centrifuged at 8000 rpm for 20 min; serial dilutions were then prepared in distilled sterile water and 150 μL of each dilution were poured in wells made on a Petri dish containing 30 mL of soft (8 g L^−1^ agar) Tryptic Soy Agar (TSA) inoculated with *Listeria innocua* (0.1% *v*/*v* of a pre‐grown culture in TSB). Plates were then incubated overnight at 37 °C. Bacteriocin activity (AU mL^‐1^) was quantified as the reciprocal of the highest dilution exhibiting a clear zone of inhibition, per milliliter, as reported elsewhere.^11^ Sakacin‐A concentration was also expressed in terms of AU mg^‐1^ of freeze‐dried crude extract.

### CNF–sakacin‐A material preparation

CNFs were acquired by Exilva Borregaard (Sarpsborg, Norway). Active mats were prepared by mixing 0.2 g of the liophilized sakacin‐A extract with 0.2 g of CNF (2 g commercial CNF suspension), adding 20 mL of deionized water. After magnetic stirring for 30 min, suspension was laid out in Petri dish and dried at 60 °C to remove water. Mats without sakacin‐A (negative controls) were also prepared by suspending 0.2 g CNF in 20 mL of deionized water.

### Materials characterization

Infrared spectra of the produced materials were recorded for neat and modified CNFs in attenuated total reflectance (ATR) mode using a Perkin‐Elmer (Waltham, Massachusset, USA) Spectrum 65. All spectra were recorded between 4000 and 600 cm^−1^, with a resolution of 4 cm^−1^ and eight scans. Fourier‐transfer infrared (FTIR) spectra shown in figures are representative of the samples.

Thermogravimetric analysis was developed using a Perkin‐Elmer (Waltham, Massachusset, USA) simultaneous thermal analyzer (STA 6000). Samples of about 30 mg were placed in a pan and tested at a heating rate of 10 °C min^−1^ from ambient temperature to 900 °C under air. All experiments were repeated at least twice.

### *In vitro* antimicrobial activity of active materials

Antimicrobial efficacy of the mats was confirmed by two *in vitro* tests.

Qualitative assessment of antimicrobial activity was carried out using *Listeria innocua* as indicator strain applying the Agar disk diffusion method. A circular portion of 20 mm diameter was placed onto *Listeria* pre‐inoculated soft TSA plates then incubated for 16 h at 37 °C. The leaching ability of the CNF only (negative control) and of the active CNF–sakacin‐A sample were determined by the formation of a clear halo of *Listeria* growth inhibition around the samples, measuring the growth inhibition area in terms of cm^2^. All experiments were repeated three times.

For quantitative assessment of the antimicrobial activity a *Listeria* cell suspension was prepared at a concentration of 5 × 10^5^ cells mL^−1^ in 20% TSB, i.e. one volume of TSB added with four volumes of sterile isotonic solution; 200 μL of this suspension were then poured onto at least two replicates of each mat sample previously weighed (0.05 g) and dry sterilized. Inoculated materials were then incubated for 24 h at 37 °C; bacteria were then resuspended by using 50 mL of neutralizing solution: lecithine (Carl Roth GmbH, Karlsruhe, Germany) 3 g L^−1^, sodium thiosulfate (Carl Roth GmbH) 5 g L^−1^, l‐histidine (Carl Roth GmbH) 1 g L^−1^, Tween‐80 (Merck K GaA) 30 g L^−1^, potassium dihydrogen phosphate buffer (Sigma‐Aldrich) 10 mL, pH 7.2 ± 0.2. Decimal dilutions series of the resulting suspension were then carried out for *Listeria* determination (CFU mL^‐1^), employing TSA as culture medium.

### Antimicrobial activity of active materials on smoked salmon

**Figure 1:**
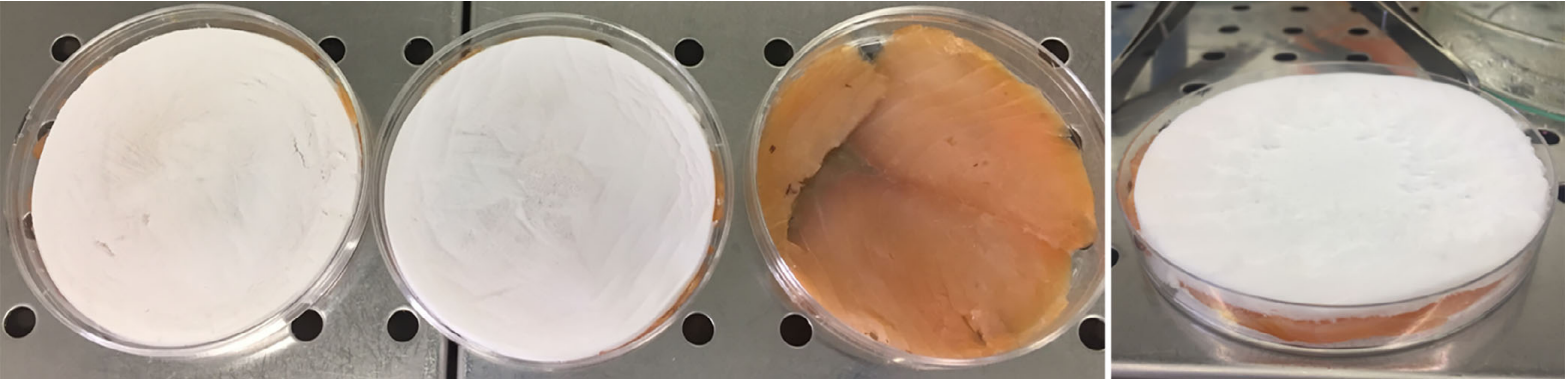
Storage trials of smoked salmon fillets: left, samples preparation in plates; right, particulars of the final assembly.

Smoked salmon fillets were purchased from a large‐scale retail channel (Vega Salmon GmbH, Handewitt, Germany, ingredient: salmon (*Salmo salar*), salt 3% on salmon weight). Fillets were aseptically cut into sections of 9 cm diameter and an area of around 60 cm^2^ and then inoculated with *Listeria innocua* cells suspension to obtain a total microbial load of approximately 10^3^ cells cm^−1^. Inoculated samples were covered (top and bottom) with CNF–sakacin‐A or CNF‐only samples (negative control) and then transferred in Petri dishes (Fig. 1).

All samples were packed in polyamide/polyethylene (PA/PE) plastic food vacuum bags (Reber, Luzzara, Italy) under vacuum and then stored at 6 °C up to 28 days. Samples of salmon fillet stored uncovered under vacuum were also prepared. Starting from t0, every 7 days salmon fillets were transferred aseptically into Stomacher bags (VWR blender bag, Milan, Italy), filled with physiological solution [9 g L^−1^ sodium chloride (NaCl; Merck K GaA), 9× sample weight) and blended in a Stomacher (Star Blender LB 400, VWR, Milan, Italy) for 3 min. Decimal dilutions series of the obtained suspension were then carried out for *Listeria* determination (CFU mL^‐1^). Selective *Listeria innocua* determination was performed employing ALOA culture medium (Agar *Listeria* Ottaviani Agosti added with enriched and selective supplements; Biolife, Milan, Italy) and plates incubated at 37 °C for 48 h. Experiments were replicated twice. Counts were reported as logarithm of the number of colony forming units (Log CFU g^‐1^ salmon), and mean and standard deviation calculated.

### Statistical analysis

Statistical analysis was performed using GraphPad Prism software (version 8.0.1, San Diego, CA, USA), the effect of two factors (time and treatment) were investigated by analysis of variance (ANOVA) according to the general linear model. When the effect was significant (*P* < 0.05), differences between means were separated by Tukey test of multiple comparisons.

## RESULTS

### Sakacin‐A production and purification

**Figure 2:**
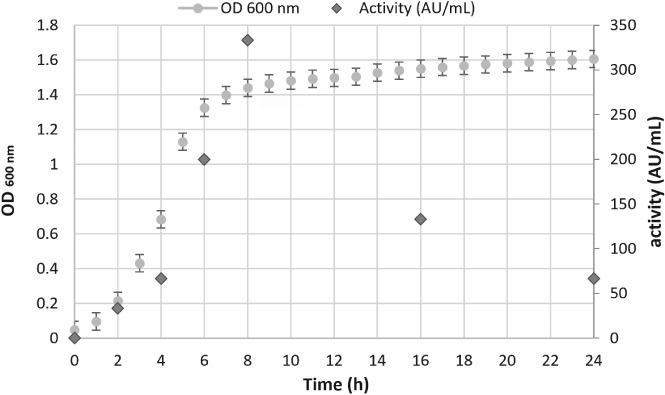
*Lactobacillus sakei* growth profile and sakacin‐A production in terms of activity (AU mL^‐1^).

*Lactobacillus sakei* in the applied culture conditions was found to grow with a lag phase of 98.29 ± 6.99 min, a final value of 1.61 OD and a maximum rate of 0.010 ± 0.001 OD min^‐1^ [*R* ^2^ = 0.987, standard error (SE) of fit = 0.056] (Fig. 2).

The highest sakacin‐A production (333 AU mL^‐1^) was achieved at the beginning of the stationary phase, around 8 h incubation, confirming that bacteriocin production takes place during the exponential phase of the microbial growth, as reported in the literature.^2^, ^28^

Based on the obtained results, a masterbatch of 7 L of *L actobacillus sakei* liquid culture was produced. After centrifugation, supernatant was added with ammonium sulfate to obtain a sakacin‐A enriched precipitate. The wet precipitate was collected in distilled water (1/10 of supernatant volume) and then freeze‐dried, obtaining 31.60 g of sakacin‐A extract (4.51 g L^−1^ of initial culture medium). The total yield of the enrichment procedure set near to a total antimicrobial activity of 530 000 AU (Table 1), that means 25% of initial activity. Although ammonium sulfate precipitation is causative of the main losses (and its implementation is a future desirable outcome), this step allows a straightforward purification of the bacteriocin, increasing the specific activity from 56 to 256 AU per mg of total protein.

**Table 1:** Sakacin‐A recovery yield

Fraction	Volume (mL)	Activity (AU mL^‐1^)	Total activity (AU)	Protein content (mg mL^−1^)	Specific activity (AU mg^‐1^ protein)	Yield (%)
Culture medium	7000	337	2 359 000	n.d.^a^	n.d.	100
Supernatant	6300	333	2 097 900	6.0	56	90
After ammonium sulfate precipitation, 10× redissolved	630	933	587 790	3.2	291	28
Sakacin‐A extract	31.60 g	16.70 AU mg^‐1^	527 720	63 mg g^−1^	265	25

a n.d., not determinable.

### Material characterization

**Figure 3:**
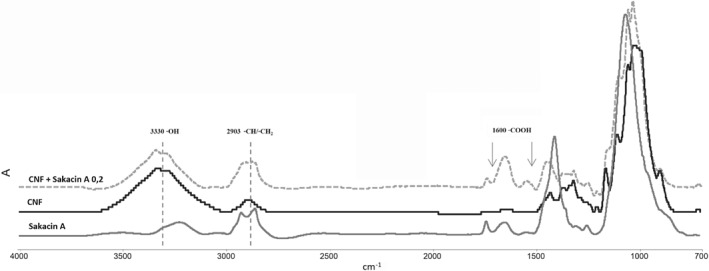
FTIR spectra of materials prepared employing cellulose nanofiber (CNF) only and CNF–sakacin‐A; spectrum of the sakacin‐A enriched extract in the same condition is also reported.

Mats were produced by mixing CNFs with the sakacin‐A enriched extract. FTIR analysis was carried out on pure sakacin‐A extract, CNF mat and CNF–sakacin‐A incorporated samples (Fig. 3). For the pure sakacin‐A extract, a strong band for the alkyl group was observed at 2903 cm^−1^ and for the carboxyl group at 1600 cm^‐1^. The peaks between 1500 and 1300 cm^‐1^ represent the presence of the nitrogen groups. Well known conventional bands for cellulose were observed at 3300, 1250–1460, 2850–2980 and 1170–1150 cm^−1^ corresponding to the stretching vibrations of hydroxyl groups (OH), alkyl groups (CH and CH_2_) and C–O–C bonds from glycosidic bridges, respectively. Sakacin‐A incorporated CNF material presented both prime peaks for the sakacin‐A extract and CNF. With the addition of sakacin‐A, a significant increase in the peak for the alkyl and the appearance of a small peak at 1600 cm^−1^ for the carboxyl group were observed confirming the incorporation of sakacin‐A.

**Figure 4:**
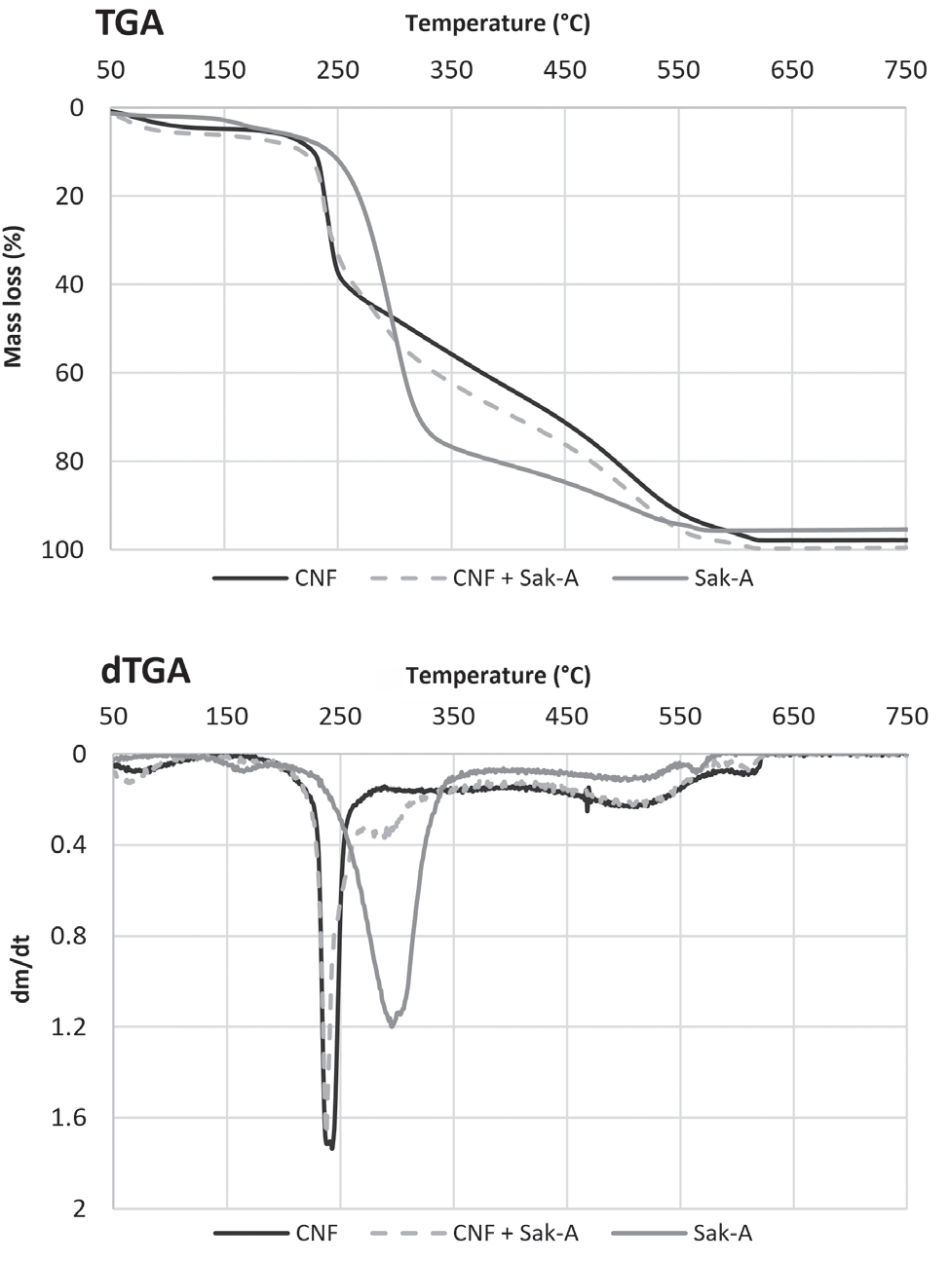
Thermogravimetric analysis of materials prepared employing cellulose nanofiber (CNF) only and CNF–sakacin‐A: a, original unit; b, first derivative. The behavior of an enriched sakacin‐A solution at the same concentration employed is also reported.

The analysis of the thermal properties of the fibers provides valuable information about the physical and chemical characteristics after incorporation of sakacin‐A extract. The thermograms (weight loss) of the sakacin‐A, neat CNFs and sakacin‐A incorporated CNFs (original values and first derivative) are shown in Fig. 4(a, b), respectively. The neat cellulose showed a conventional thermogram with maximum degradation temperature at around 250 °C,^29^ with a weight loss of 5% to 7% below 150 °C due to the residual moisture stored in the neat CNF material. The sakacin‐A extract was also examined and demonstrated a maximum degradation profile at around 300 °C. With the incorporation of sakacin‐A in CNFs, even if thermograms showed degradation profiles similar to the CNF‐only sample, differential thermogravimetric analysis profiles evidenced the presence of the sakacin A signal.

### *In vitro* antimicrobial activity

**Figure 5:**
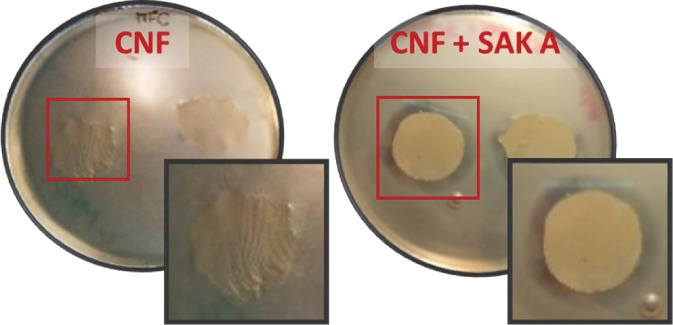
Solid Tryptic Soy Agar (TSA) culture of *Listeria innocua* grown at 37 °C in presence of cellulose nanofiber (CNF)‐alone (negative control, left) or with CNF‐sakacin‐A (antimicrobial sample, right) materials.

Trials carried out *in vitro* with solid cultures of *Listeria innocua* confirmed that CNF–sakacin‐A mats possess antimicrobial activity, evident with the formation of 6.7 ± 0.8 cm^2^ halo of growth inhibition onto *Listeria*‐agar plates. In contrast, CNF‐alone products prepared without the bacteriocin (negative control) did not produce any growth inhibition (Fig. 5).

**Figure 6:**
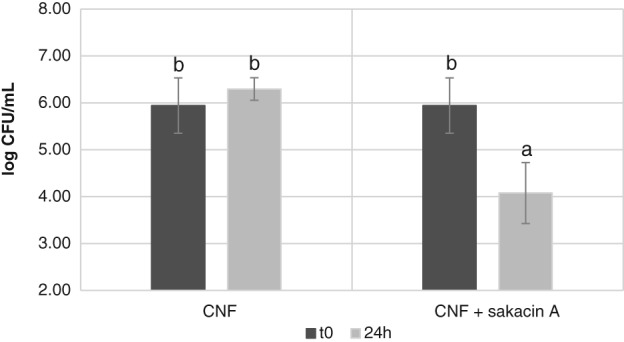
*Listeria innocua* cell concentration (in terms of Log CFU mL^‐1^) in cultures incubated at 37 °C for 24 h in presence of CNF‐alone (negative control, left) or with CNF‐sakacin‐A (antimicrobial sample, right) materials.

Quantitative trials performed to determine the capacity of the produced active materials to reduce the population present in a *Listeria* cell suspension evidenced a 2‐log cycle reduction (from 6 to 4 Log CFU mL^‐1^) when the active material was kept in contact for 24 h (Fig. 6).

Note that *Listeria innocua* was taken as surrogate of the food‐borne pathogenic *Listeria monocytogenes*. A surrogate is a bacterium that has physiological characteristics nearly identical to a pathogenic bacterium of interest, and is used to give a margin of safety to the researchers and to prevent unnecessary exposure to pathogens.^30^

### *In vivo* antimicrobial activity

CNF–sakacin‐A active mats were evaluated for their antimicrobial effectiveness on food. *Listeria* population was determined in samples of smoked salmon purposely contaminated with *Listeria innocua* and then stored at 6 °C for up to 28 days (Table 2).

**Table 2:** *Listeria* population (Log CFU g^‐1^) in samples of smoked salmon intentionally inoculated and then stored for up to 28 days at 6 ± 1 °C in absence as well as in presence of the cellulose nanofiber (CNF)‐only or the active CNF‐sakacin‐A mats

	Salmon + *Listeria innocua*			Salmon + *Listeria innocua* + CNF			Salmon + *Listeria innocua* + CNF‐sakacin‐A		
Sample Days	Log CFU g^‐1^	Standard deviation	Growth value	Log CFU g^‐1^	Standard deviation	Growth value	Log CFU g^‐1^	Standard deviation	Growth value
0	3.99 ^b^	0.08	0.00	3.99 ^b^	0.08	0.00	3.99 ^b^	0.08	0.00
7	5.38 ^c^	0.06	+1.39	5.32 ^c^	0.13	+1.34	3.22 ^a^	0.19	−0.76
14	6.84 ^e^	0.06	+2.85	7.29 ^e^	0.14	+3.30	4.08 ^b^	0.30	+0.09
21	8.74 ^f^	0.12	+4.75	8.20 ^f^	0.14	+4.21	5.90 ^cd^	0.11	+1.91
28	8.90 ^f^	0.08	+4.91	8.70 ^f^	0.29	+4.71	6.08 ^d^	0.13	+2.09

Means with different superscript letters are different (*P* < 0.05).

Results evidenced a 2‐log cycles reduction of *Listeria* population in salmon samples stored in the presence of CNF–sakacin‐A mats, with respect to the population inoculated at t0 (6.08 *versus* 3.99 Log CFU g^‐1^). Moreover, when comparing results with the other trials performed without or with CNF‐only film, the final population was reduced by about 2.5–3 Log cycles (8.70–8.90 Log CFU g^‐1^).

These experiments demonstrate the antimicrobial activity of the CNF–sakacin‐A active material and their effectiveness against *Listeria innocua* in storage trials of a ready‐to‐eat food product.

## DISCUSSION

The present research focused on developing and characterizing a novel cellulose‐based antimicrobial material employing a freeze‐dried enriched preparation of the bacteriocin sakacin‐A. In the first step the bacteriocin was produced in liquid culture employing a low‐cost medium formulated with cheese whey permeate (CWP), a residual by‐product obtained by extraction of whey proteins from cheese whey by ultrafiltration. While for both whey protein and cheese whey official statistical data on production and market value are available – Eurostat for the European Union (EU)^31^ and Clal for Italy^32^ – no official figures can be found for CWP. Even if such lack of data shows its low value for standard economic activities, CWP production is pulled by the highly profitable activity of whey protein extraction. In 2017 liquid cheese whey available was 48 million of tonnes for EU‐28, with an increase of 12% over the last 10 years. In this frame, CWP may represent a cheap and highly available substrate for circular economy activities.^33^

As regards sakacin‐A purification, in the present article an ammonium sulfate precipitation was applied to the culture supernatant, allowing an enriched bacteriocin extract containing 16.7 AU mg^‐1^ of the active compound to be obtained, with a total activity yield of 25%. Purification is an essential step for bacteriocin applications but also represents a time‐consuming and a low‐yield step of the entire production process, as confirmed by the obtained data.^10^ Barbiroli *et al.* 10 reported on sakacin‐A purification from a food‐grade medium by one‐step diafiltration, giving a freeze‐dried enriched sakacin‐A with an antimicrobial titer of 1.36 AU mg^‐1^ and a total activity yield of 20%. Trinetta *et al.* 34 recovered concentrated sakacin‐A by ultra‐filtration through a 3 kDa mol exclusion membrane, lyophilized overnight and stored at 4 °C for further experiments; 1 mg of the lyophilized powder was resuspended in 1 mL of sterile water and showed an antimicrobial titre of 1600 AU mL^‐1^; however, no data about process yields were reported. Guyonnet *et al.* 35 carried out an alternative bacteriocin purification procedure based on cation exchange chromatography with a yield of 10% of pure bacteriocin, probably due to the hydrophobic characteristics of this molecule. In general, the low bacteriocin production yields and the need to combine several purification procedures (extraction, precipitation, ultrafiltration, chromatographic and molecular methods) represent the limiting step for bacteriocin application at an industrial scale.^3^, ^36^ The results obtained in this study could be considered a good starting point for further improvements in terms of bacteriocins recovery yields.

In the present article, sakacin‐A extract was combined with CNF to produce an antimicrobial cellulosic material. To the best of our knowledge, this is the first study related to sakacin‐A and CNF combination for the formation of an antimicrobial material. Cellulose shows interesting properties to produce biodegradable films as well as for its application as a carrier of antimicrobial molecules. Different strategies for the conjugation or grafting of antimicrobial compounds on CNF have been described in the literature but their efficacy is closely connected to the active molecule (aminosilanes, antibiotics, bacteriocins, *etc*.).^17^, ^23^, ^37^

The set‐up of the CNF–sakacin‐A active materials was found effective in reducing *Listeria* population in samples of smoked salmon of about 3 Log cycles with respect to the values reached without or with CNF‐only samples after 28 days of storage. In the applied conditions, the bacteriocin concentration in mats was around 50 AU cm^−2^. Barbiroli *et al.* 10 reported on the development of a sakacin‐A active paper (0.63 mg cm^−2^) produced by coating PE‐coated paper sheets employing a crude sakacin‐A extract to obtain an active antimicrobial package. Storage trials of thin‐cut veal meat slices inoculated with *Listeria* laid on active paper sheets evidenced a 1.5 Log units reduction of *Listeria* population with respect to control after 48 h at 4 °C. Trinetta *et al.* 34 demonstrated the efficacy of the same bacteriocin against different epidemic clones of *Listeria monocytogenes* in pullulan films: experimentally inoculated surfaces of turkey breast were covered with a section of sakacin‐A‐containing (1 mg cm^−2^) pullulan films, and the results showed a reduction of up to 3 Log CFU g^‐1^ after 3 weeks under refrigerated storage.

Literature data related to active materials produced with bacteriocins are often referred to as nisin, due to the fact that to date it is the only bacteriocin approved by the Food and Drug Administration (FDA) and by the EU. Generally, nisin is incorporated into the polymer via direct and simple blending, as reported by Coma *et al.* 14 and by Imran *et al.* 16 that combined nisin and hydroxypropylmethylcellulose by incorporating the bacteriocin into the film‐forming solution prior to film formation, however this strategy presents limitation and shows disadvantages in maintaining the antimicrobial activity for long terms. Different possible solutions were shown by Saini *et al.* 17 that covalently linked nisin on the surface of TEMPO oxidized CNFs for food packaging application, and by Wu *et al.* 38 that developed a green process of anchoring nisin onto oxidized cellulose, exploiting the interaction between the amino group of nisin and the aldehyde group present on oxidized cellulose.

From an overall look at the obtained results, the developed CNF‐sakacin A material, not intended as a way to ‘clean’ a contaminated food product, can significantly contribute to reduce the risk of *Listeria monocytogenes* outbreaks, especially when used as mats or absorbant pads within slices of ready‐to‐eat food products.

## CONCLUSIONS

This work aimed at creating a sustainable and active anti‐listerial material, in which a protein extract containing the bacteriocin sakacin‐A was produced and then absorbed onto CNFs in the absence of chemical modifications. Incorporation of the antimicrobial was demonstrated, as well as its antimicrobial activity. Application of CNF‐sakacin‐A mats on smoked salmon fillets under conditions similar to those foreseeable for a future practical use proved the antimicrobial activity against *Listeria*. Future trials will be aimed at investigating whether this antimicrobial material may find practical uses (e.g. paper liners or wraps) and its effectiveness when in contact with other food products, taking into account that antimicrobial release and activity may vary depending on the nature, the composition and humidity of food items.
